# 

**Published:** 1873-05

**Authors:** 


### TABLE OF CONTENTS.

ORIGINAL COMMUNICATIONS.

Functional Diseases of the Eye—Hypermetropia. By Swan M. Burnett, M.D. 257
Eclampsia Infantum. By T. Curtis Smith, M.D. . . . . . . 267
Congestive Malarial Fever. By Benjamin Rhett, M.D. . . . . . 273

CORRESPONDENCE.

Uterine Hemorrhage. By John H. Weir, M.D. . . . . . . 279
Meeting of the Mississippi State Medical Association. By S. V. D. Hill, M.D. 282

EXTRACTS AND GLEANINGS.

Clinical Significance of Sputa—285. The Treatment of Prolapsus Uteri Without Mechanical Means—287. The Extraction of Foreign Bodies from the External Ear—288. Diagnosis of Tuberculosis—290. Hooping-Cough—291. Rhus Toxicodendron—292. Belladonna in Ophthalmia—293. Ergotine as a Hemostatic—294. Spina-Bifida—295. Ergot in the Treatment of Cystitis with Hematuria—296. Sick Headache—297. Formulæ; Ingrowing Nail—298. Hydrophobia; Typho-Malarial Fever—299. Opium; To Prevent Pitting in Small-Pox—300. Apomorphine as an Emetic; Sulphate of Cinchona; Tracheotomy—301. Sulpho-Vinate of Soda; Puncture of the Abdomen for the Relief of Tympanitis; Nitrate of Silver in Epilepsy and Chorea—302. Balsam of Copaiba in Small-Pox and Scarlatina; Posture as an Important Adjunct in the Treatment of Diarrhœa and Dysentery; Treatment of Chilblains—303. A Good Lesson on Diagnosis; Pills of Ferrous Oxide; The Local and Superficial Anæsthetic Effects of the Phenols—304. Preparations of Eucalyptus Globulus; Sulphate of Iron in Suppuration; Cancer of the Liver; Tincture of Calendula in Phagedæna—305. Treatment of the Inflated Breasts of Nurses; Purification of Chloral Hydrate; Use of Camphor in Hospital Gangrene; Method of Making Leeches Bite—306. Tincture of Iodine in Cholera Infantum and Dysentery; Treatment of Enlarged Tonsils; The Therapeutic Value of Gelseminum; Treatment of Gastralgia—307. Lactic Acid in Dyspepsia; Treatment of Psoriasis; Nux Vomica in the Diarrhœa of Exhaustion—308—Treatment of Gonorrhœa by Tanno-Glycerin Paste; Phtheriasis; Carbolic Acid in Obstinate Leucorrhœa; Citrate of Iron and Sulphate of Quinine in Chlorosis and other Anæmic States—309. Foreign Body in Interior of Eye; Dugong Oil; Nocturnal Incontinence Treated by Chloral; External Use of Fuming Nitric Acid—310. Typhoid Fever; Arsenic in Disease of the Liver; Tincture of Chloride of Iron for Corns; Extracts in Powder; Erysipelas; Vermifuge Sirup—311. A New Mode of Administering Copaiba; The Endermic Application of Belladonna for Neuralgia; Strychnia in Albuminuria; Urticaria from Privy Seats; Nitrite of Amyl in Spasmodic Asthma; Pill for Gastralgia—312.

EDITORIAL AND MISCELLANEOUS.

Our Terms, Cash; Communications; Texas Medical College, . . . 313
Kind Words and Suggestions; Texas Medical Association, . . . 314
From One of Our Co-Laborers, . . . . . . . . . . . 316
An Honor Worthily Bestowed; The Obstetrical Journal; List of Payments to the RECORD, Commencing with the January Number, . . . 319

